# IL-6 secretion in osteoarthritis patients is mediated by chondrocyte-synovial fibroblast cross-talk and is enhanced by obesity

**DOI:** 10.1038/s41598-017-03759-w

**Published:** 2017-06-14

**Authors:** Mark J. Pearson, Dietmar Herndler-Brandstetter, Mohammad A. Tariq, Thomas A. Nicholson, Ashleigh M. Philp, Hannah L. Smith, Edward T. Davis, Simon W. Jones, Janet M. Lord

**Affiliations:** 1 MRC-Arthritis Research UK Centre for Musculoskeletal Ageing Research, Institute of Inflammation and Ageing, University of Birmingham, West Midlands, B15 2TT UK; 2 Department of Immunobiology, Yale School of Medicine, New Haven, CT 06520 USA; 3 Royal Orthopaedic Hospital, Bristol Road South, Birmingham, West Midlands B31 2AP UK

## Abstract

Increasing evidence suggests that inflammation plays a central role in driving joint pathology in certain patients with osteoarthritis (OA). Since many patients with OA are obese and increased adiposity is associated with chronic inflammation, we investigated whether obese patients with hip OA exhibited differential pro-inflammatory cytokine signalling and peripheral and local lymphocyte populations, compared to normal weight hip OA patients. No differences in either peripheral blood or local lymphocyte populations were found between obese and normal-weight hip OA patients. However, synovial fibroblasts from obese OA patients were found to secrete greater amounts of the pro-inflammatory cytokine IL-6, compared to those from normal-weight patients (p < 0.05), which reflected the greater levels of IL-6 detected in the synovial fluid of the obese OA patients. Investigation into the inflammatory mechanism demonstrated that IL-6 secretion from synovial fibroblasts was induced by chondrocyte-derived IL-6. Furthermore, this IL-6 inflammatory response, mediated by chondrocyte-synovial fibroblast cross-talk, was enhanced by the obesity-related adipokine leptin. This study suggests that obesity enhances the cross-talk between chondrocytes and synovial fibroblasts via raised levels of the pro-inflammatory adipokine leptin, leading to greater production of IL-6 in OA patients.

## Introduction

Osteoarthritis (OA) is a progressive age-related degenerative joint disease, which culminates in the loss of articular cartilage mass, remodelling of the subchondral bone and joint space narrowing^1^. Currently the only treatments for this debilitating condition are analgesics and eventually join replacement. Inflammation is increasingly being recognised as a driver of OA disease pathology thus implicating the synovial environment, including the role of inflammatory cytokines and infiltrated immune cells, in driving the degeneration of cartilage tissue^2–4^. Indeed, synovitis has been demonstrated in OA patients histologically and by MRI and ultrasound imaging^5, 6^. However, not all OA patients exhibit overt signs of synovitis, indicating that the involvement of inflammation and immune cells in the disease pathology is dependent on the patient. Furthermore, if this is the case the treatment of OA patients should not be uniform and the inflammatory phenotype should be used in patient stratification.

One contributing factor in inflammatory OA may be the adiposity of the patient, since it is now known that adipose tissue is an active endocrine organ, which can secrete pro-inflammatory cytokines (adipokines) that can affect joint integrity. For example, *in vitro* studies on the adipokines leptin and visfatin have implicated particular adipokines in mediating cartilage degradation^7–10^ and synovial inflammation^11, 12^. Importantly, obesity is associated with OA in both weight-bearing and non-weight bearing joints^13, 14^, suggesting it is not simply due to increased load bearing. Indeed, previous studies have shown that obese adipose tissue is more inflammatory with increased infiltration of macrophages and increased secretion of pro-inflammatory adipokines^15–17^.

We hypothesised, therefore, that obese patients with OA would exhibit a differential synovial fluid cytokine expression compared to normal-weight OA patients and that this would be accompanied by differences in local and/or systemic immune lymphocyte populations.

In this study, we have characterised the cytokine profile of synovial fluid and profiled bone marrow (isolated from the arthritic hip) and peripheral blood T cell subsets from obese and normal-weight OA patients, and conducted cellular cross-talk studies between chondrocytes and fibroblasts to specifically elucidate differences in pro-inflammatory signalling by IL-6.

## Results

### IL-6, IL-8 and leptin are elevated in the synovial fluid of obese OA patients

**Figure 1:**
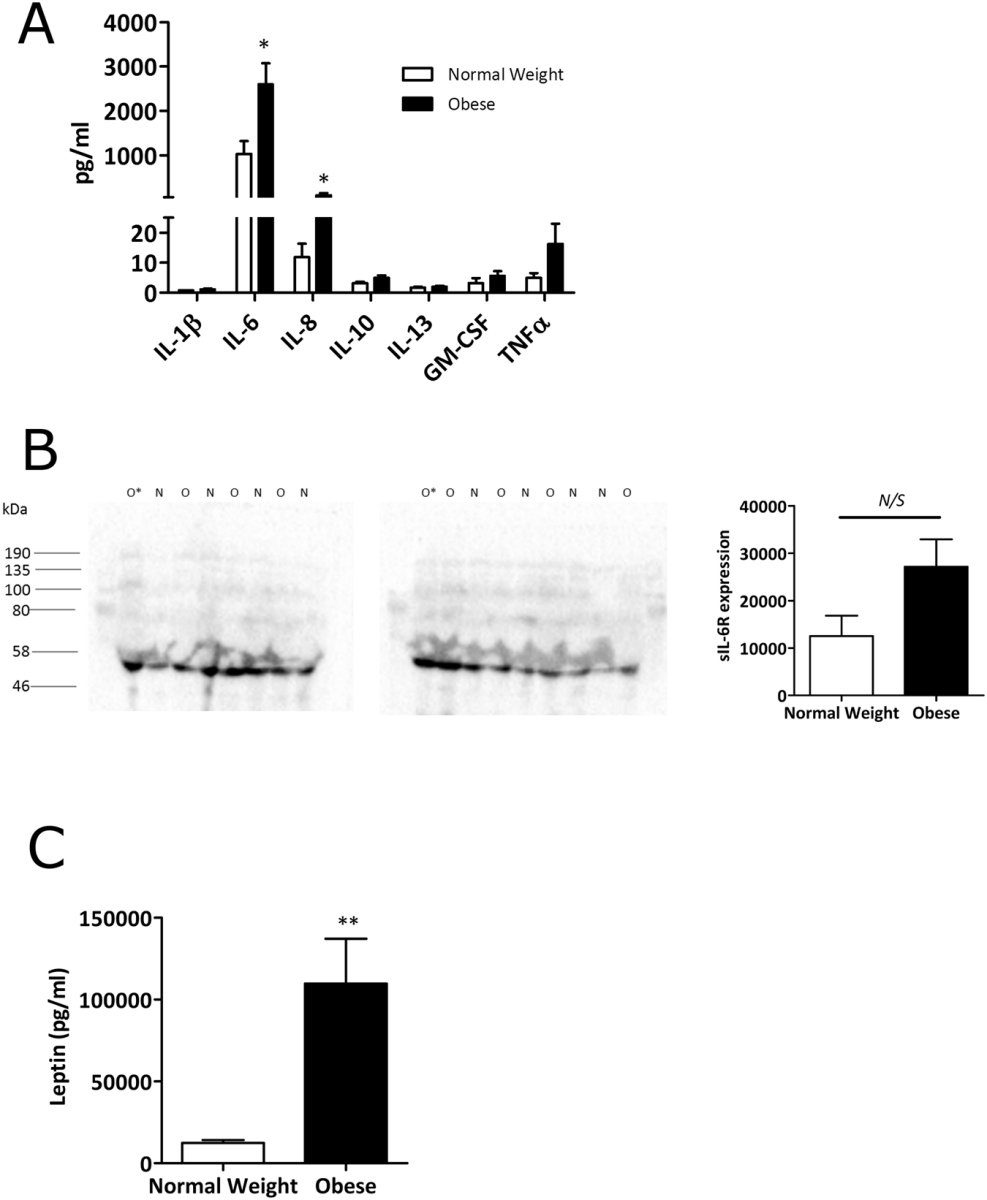
Synovial fluid from obese OA patients contains greater amounts of IL-6 and IL-6R than synovial fluid from normal-weight OA patients. (**A**) Bio-plex assay data showing the profile of inflammatory markers present in the synovial fluid of normal-weight (n = 8) and obese (n = 8) OA patients. (**B**) sIL-6R expression in n = 8 normal-weight (N) and n = 8 obese (O) OA synovial fluid samples was determined by Western blotting and densitometry loaded for equal total protein content (20 µg). O* represents an obese sample that was loaded onto both blots for standardisation. (**C**) Bio-plex assay data showing leptin concentration in the synovial fluid of normal-weight (n = 19) and obese (n = 27) OA patients. Data are mean ± SEM. Statistical significance was determined by Mann-Whitney U test. *p < 0.05, **p < 0.01, N/S = not significant.

We first compared the inflammatory phenotype of obese and normal-weight OA patients by profiling the concentrations of a panel of pro-inflammatory cytokines in the synovial fluids of their affected joint. Importantly, this analysis revealed that synovial fluid from obese OA patients contained significantly higher concentrations of both IL-6 and IL-8 (p < 0.05) (Fig. 1A). In addition, synovial fluid from obese OA patients contained approximately 3-fold greater amounts of TNFα than normal-weight synovial fluid, albeit this did not reach statistical significance (Fig. 1A).

Since IL-6 was found to be the most abundantly elevated cytokine in obese synovial fluid and is a proximal driver of inflammation, we then investigated this further by profiling the protein expression of the soluble IL-6 receptor (sIL-6R) in both obese and normal-weight synovial fluid by Western blotting. Of note, synovial fluid from obese OA patients also exhibited greater amounts of sIL-6R expression compared to normal-weight. However levels were highly variable across patients and as such these data did not reach significance (Fig. 1B).

Given the observed increases in pro-inflammatory cytokines in synovial fluid from obese OA patients, we wondered whether adipokines were also highly expressed in synovial fluid from these patients. To determine this, synovial fluid samples from normal weight (n = 19) and obese (n = 27) patients were assessed in a Luminex assay. We found that leptin expression was significantly elevated in synovial fluid from obese OA patients (p < 0.01) (Fig. 1C).

### Obese patients with hip OA do not exhibit different T cell subsets in the peripheral blood or in the bone marrow, compared to normal-weight patients

We next examined whether the normal-weight and obese synovial fluid cytokine profiles reflected differences in T lymphocyte populations. To this end, both blood and bone marrow samples were collected from both normal-weight (n = 12) and obese (n = 12) OA patients, and following PBMC isolation, different T cell subsets were discriminated via immunostaining and flow cytometry using a range of well-characterised surface and intracellular markers.

No statistically significant differences could be observed in CD4:CD8 ratios in either the peripheral blood or bone marrow (from the arthritic hip) between normal-weight and obese OA patients (Table 1). Normal-weight and obese OA patients also did not show any differences in the frequency of CD4^+^ or CD8^+^ T cell subsets, such as naïve (T_N_), central-memory (T_CM_), effector-memory (T_EM_) and CD45RA-expressing effector-memory (T_EMRA_). In contrast to rheumatoid arthritis, highly differentiated CD28^−^ T cells did not accumulate in the blood or bone marrow of obese OA patients. There were also no differences in the frequency of regulatory CD4^+^ T cells (Treg) or activated/tissue-resident CD69^+^ T cells between normal-weight and obese OA patients. The percentage of cytokine-expressing CD4^+^ and CD8^+^ T cells was not different between normal-weight and obese OA patients.

**Table 1:** The impact of obesity on T cell subsets in the peripheral blood and bone marrow of OA patients.

	Normal weight (BMI 18.5–24.9)		Obese (BMI ≥ 30.0)	
	PB	BM	PB	BM
CD4:CD8 ratio	2.7 ± 0.8	0.7 ± 0.1	2.9 ± 0.6	1.4 ± 0.3
***CD4*** ^**+**^ ***T cells***				
% T_N_	45.5 ± 6.6	25.8 ± 5.8	43.3 ± 3.8	31.4 ± 4.3
% T_CM_	34.3 ± 3.6	31 ± 5	40.9 ± 3.2	33.7 ± 2.7
% T_EM_	14.1 ± 4.3	38.9 ± 4.8	13.9 ± 1.8	32.3 ± 3.4
% T_EMRA_	5.4 ± 1.2	4.3 ± 1	3.3 ± 1.2	2.7 ± 0.4
% CD28^−^	1.5 ± 0.6	1.7 ± 0.4	1.1 ± 0.4	1.3 ± 0.4
% CD69^+^	0.2 ± 0.1	15.3 ± 1.9	0.4 ± 0.2	15.2 ± 2.5
% T_reg_	3 ± 0.4	3.3 ± 0.7	3.5 ± 0.9	3 ± 0.4
% IFN-γ^+^	19.2 ± 3.4	31.7 ± 6	10.1 ± 1.9	21.2 ± 6.2
% IL-4^+^	3.7 ± 1.1	3.4 ± 0.9	2.4 ± 0.7	1.6 ± 0.7
% IL-2^+^	59.7 ± 5.1	47.4 ± 8.7	55.1 ± 5.7	32.5 ± 8.3
% IL-10^+^	0.38 ± 0.06	1.6 ± 0.6	0.29 ± 0.05	1.7 ± 0.2
% IL-17A^+^	0.34 ± 0.11	0.67 ± 0.32	0.77 ± 0.12	1.14 ± 0.16
% IL-17A^+^ IFN-γ^+^	0.01 ± 0.004	0.14 ± 0.06	0.09 ± 0.05	0.39 ± 0.08
% polyfunctional	10.9 ± 1.3	19.8 ± 4.2	6.3 ± 1.3	10.8 ± 6.6
***CD8*** ^**+**^ ***T cells***				
% T_N_	14.8 ± 5.5	6.3 ± 0.9	12.3 ± 2.4	8.6 ± 1.3
% T_CM_	15.3 ± 2.6	12.3 ± 3.9	19.9 ± 3.9	14 ± 1.7
% T_EM_	37.2 ± 6.1	51.6 ± 2.4	38.2 ± 7.2	50.5 ± 3.9
% T_EMRA_	32.7 ± 4.5	29.7 ± 5	29.6 ± 4.6	26.9 ± 3.4
% CD28^−^	46.7 ± 9.8	32.5 ± 4.1	43.2 ± 10.5	32.3 ± 6.1
% CD28^−^ CD57^+^	28.1 ± 10.8	6.5 ± 3.1	33.5 ± 8.9	11.5 ± 4.9
% CD69^+^	1.1 ± 0.3	44.4 ± 5.8	1.5 ± 0.3	52.1 ± 5.6
% IFN-γ^+^	47.8 ± 10.4	54 ± 9.6	49.7 ± 9.5	64.2 ± 11.3
% IL-2^+^	22.1 ± 6.6	21.4 ± 7.1	19.6 ± 5.5	22.1 ± 7.6
% polyfunctional	13.9 ± 2.8	19.3 ± 4.3	11 ± 3.5	22.9 ± 1.6

Data are expressed as mean ± SEM. T_reg_ cells were defined as CD25^hi^ CD127^dim^ cells. Polyfunctionality was defined as cells that simultaneously produced IFN-γ, TNF-α and IL-2. PB = peripheral blood; BM = bone marrow.

### Synovial fibroblasts from obese OA patients secrete higher amounts of IL-6

**Figure 2:**
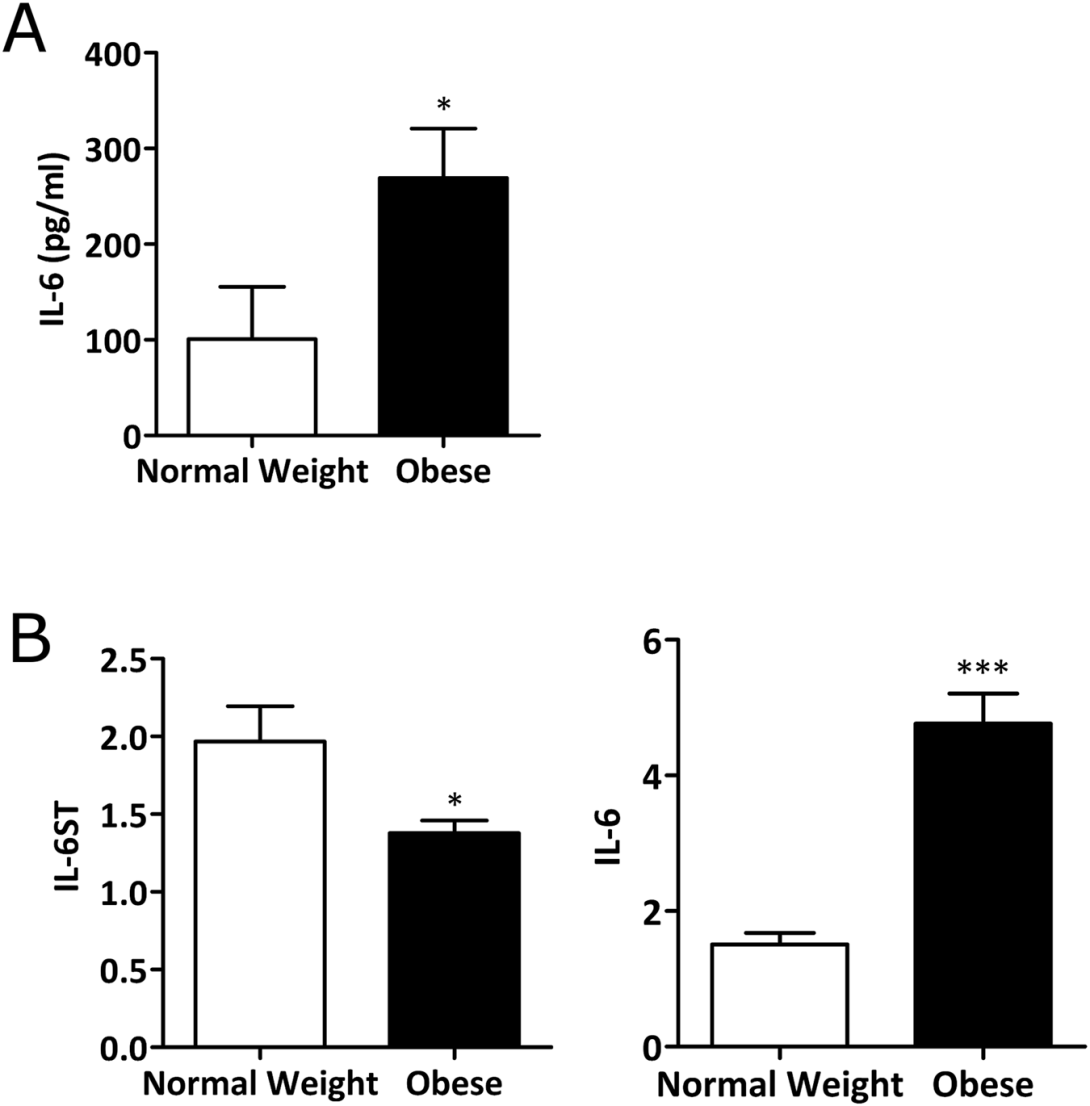
Synovial fibroblasts from obese OA patients secrete more IL-6 than cells from normal weight patients. (**A**) Secretion of IL-6 at baseline from normal weight (n = 4) and obese synovial fibroblasts (n = 4), as measured by ELISA. Data are mean ± SEM *p < 0.05. (**B**) mRNA expression of IL-6ST (gp130) and IL-6 as determined by quantitative real-time PCR. Data are mean ± SEM (normal weight n = 6; obese n = 12). Statistical significance was determined by Mann-Whitney U test where *p < 0.05, and ***p < 0.001.

Given the greater concentration of IL-6 in the synovial fluid of obese OA patients, we next analysed whether this was likely to reflect differences in the phenotype of the synovial fibroblasts. To this end, synovial fibroblasts were cultured from normal-weight and obese OA patients, and both cell supernatants and cell lysates analysed for the expression of IL-6 signalling components. We found that synovial fibroblasts from obese OA patients secreted greater amounts of IL-6 than synovial fibroblasts from normal-weight OA patients (Fig. 2A), which was also reflected in a 3-fold higher expression of IL-6 mRNA (p < 0.01) (Fig. 2B). This suggested that the obese synovial fibroblasts are imprinted with a greater inflammatory phenotype. Of note, expression of the membrane bound gp130 (the IL-6 signalling transducer; IL-6ST) was found to be higher (1.3 fold) in fibroblasts from normal-weight OA patients (Fig. 2B).

### Chondrocyte cross-talk induces IL-6 secretion in synovial fibroblasts

**Figure 3:**
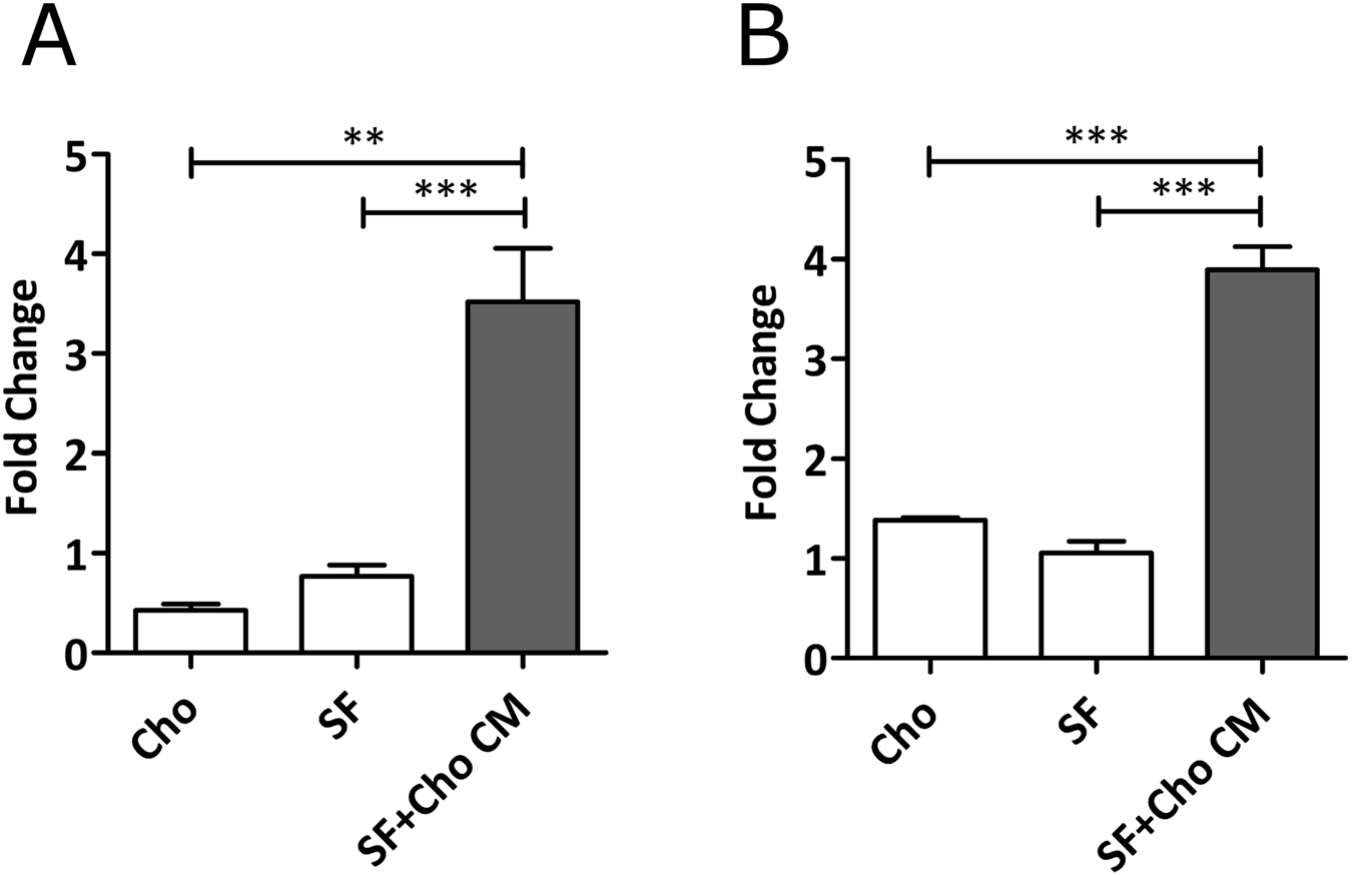
Cross talk with chondrocytes induces greater IL-6 secretion from synovial fibroblasts. (**A**) 24 h secretion of IL-6 from either chondrocytes (Cho), synovial fibroblasts (SF) or synovial fibroblasts from normal weight OA patients cultured in chondrocyte conditioned media. **(B)** 24 h secretion of IL-6 from either chondrocytes (Cho), synovial fibroblasts (SF) or synovial fibroblasts from obese OA patients cultured in chondrocyte conditioned media Data are mean ± SEM. Statistical significance was determined by one-way ANOVA **p < 0.01 ***p < 0.001.

OA is now widely accepted as a disease of the whole joint. Therefore, we next examined whether cross-talk between chondrocytes and fibroblast would affect the secretion of IL-6 from synovial fibroblasts. To this end, synovial fibroblasts were cultured for 24 h either in their own growth media or in the presence of chondrocyte conditioned media. Significantly, synovial fibroblasts from normal weight and obese OA patients that were cultured in chondrocyte conditioned media showed a statistically significant 8.2-fold and 2.8-fold increase respectively secretion of IL-6 (p < 0.001) (Fig. 3), compared to either fibroblasts (p < 0.001) or chondrocytes (p < 0.01, Fig. 3A, p < 0.001, Fig. 3B) cultured in their own growth media.

**Figure 4:**
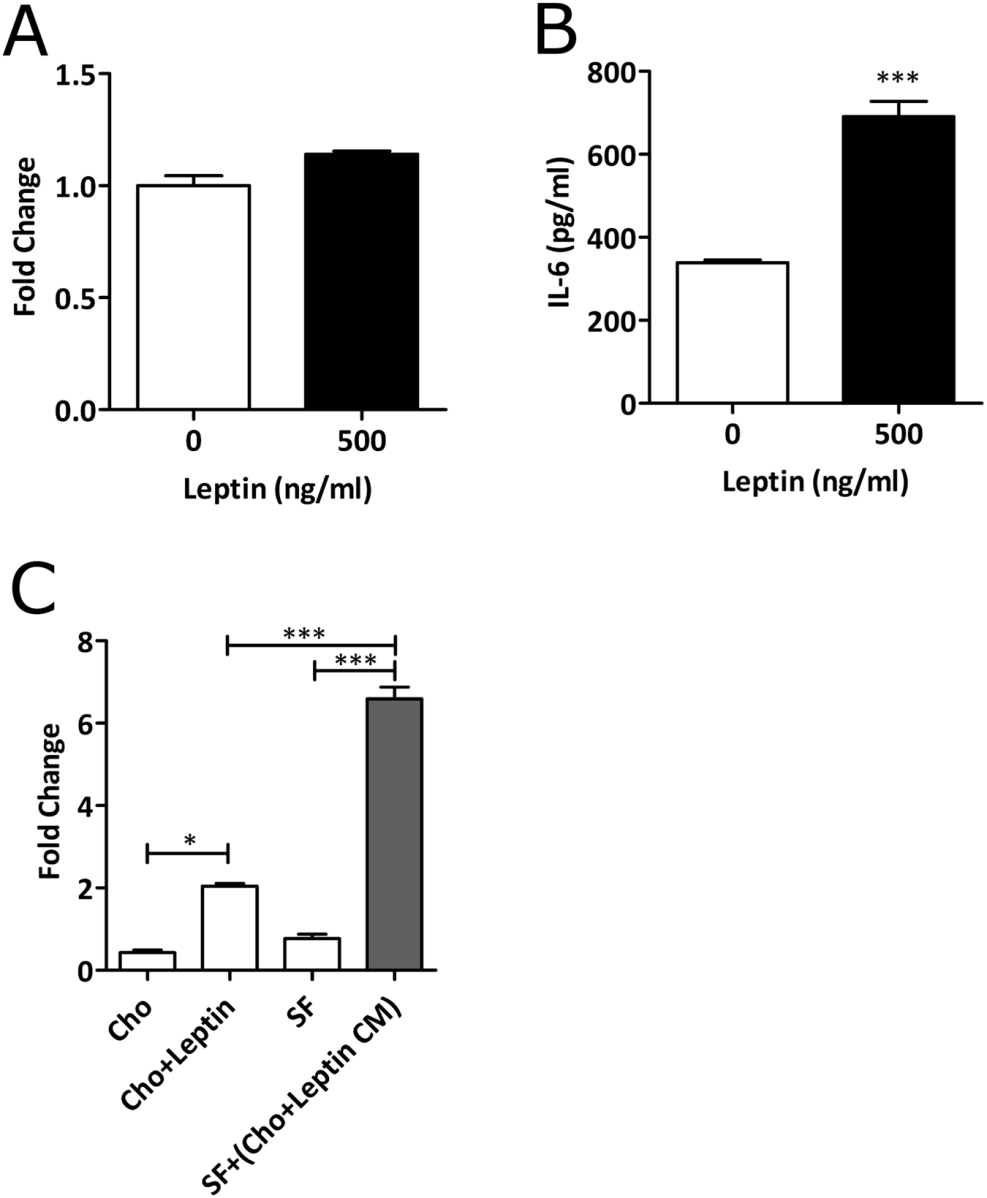
Leptin has no effect on IL-6 secretion from synovial fibroblasts, but does affect IL-6 secretion from chondrocytes. (**A**) Secretion of IL-6 in synovial fibroblasts following stimulation with 500 ng/ml leptin. (**B**) Secretion of IL-6 from chondrocytes stimulated with 500 ng/ml leptin. (**C**) Synovial fibroblasts cultured in leptin-stimulated chondrocyte conditioned media show enhanced IL6 secretion. IL6 expression was determined by ELISA. Data are mean ± SEM. Statistical significance was determined by Mann-Whitney U test and One-Way ANOVA where *p < 0.05, ***p < 0.001.

Since it is well documented that obesity is accompanied by increased production of the pro-inflammatory adipokine leptin^18–21^, we used leptin stimulation as a model to assess whether obesity was in part responsible for imprinting the obese OA synovial fibroblasts with a more inflammatory phenotype by directly stimulating IL-6 production. To this end, synovial fibroblasts from normal weight and obese patients were stimulated with leptin (500 ng/ml) for 24 h and IL-6 secretion measured by ELISA. However, compared to non-stimulated synovial fibroblasts, there was no increase in IL-6 secretion upon stimulation with leptin in either normal-weight or obese synovial fibroblasts (Fig. 4A). Conversely, leptin stimulation of chondrocytes significantly elevated IL-6 secretion (p < 0.001) (Fig. 4B). Further, analysis of the chondrocyte secretome following leptin stimulation showed that secretion of other pro- and anti-inflammatory cytokines remained unchanged (Supplementary Table S1). Of note, stimulation of chondrocytes with leptin (500 ng/ml) for 24 h elicited no induction of cellular senescence (Supplementary Fig. 3).

Given this observation, we next examined whether conditioned media from leptin-stimulated chondrocytes (containing high levels of IL-6) would induce an even greater secretion of IL-6 from synovial fibroblasts, which would be indicative of IL-6 trans-signalling. Culture synovial fibroblasts in the presence of leptin-stimulated chondrocyte conditioned media led to greater secretion of IL-6 (2.4-fold increase), compared to either fibroblasts cultured in non-leptin stimulated chondrocyte conditioned media (p < 0.001) (Fig. 4C).

**Figure 5:**
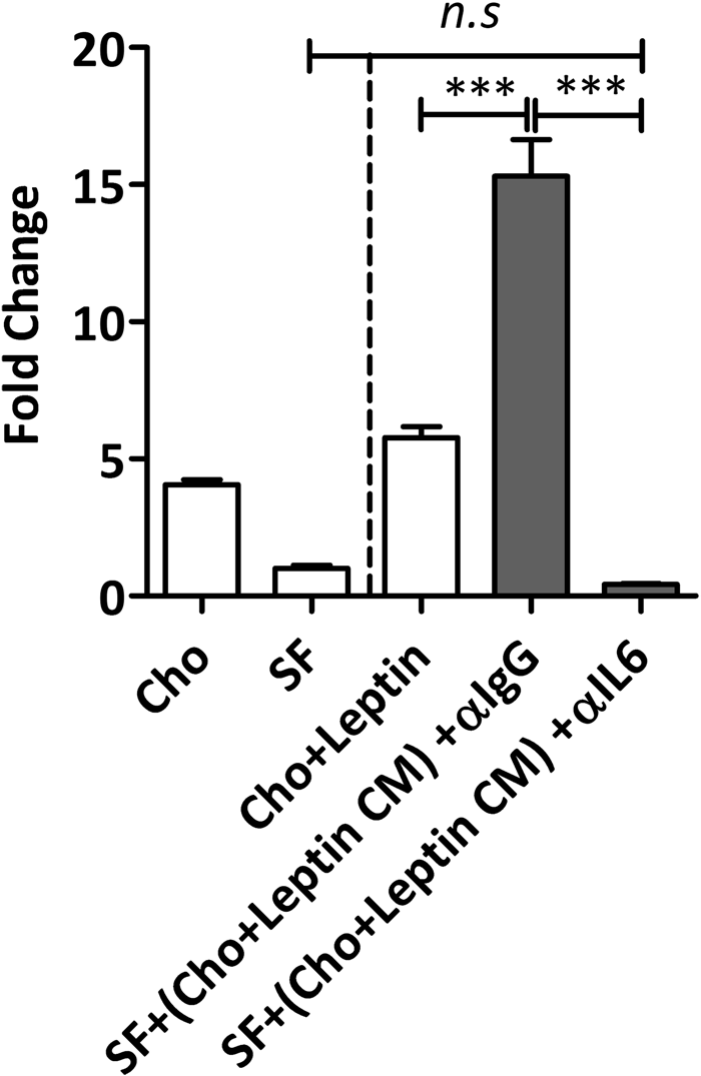
Depletion of IL-6 from chondrocyte conditioned media ablates IL-6 secretion from synovial fibroblasts. Synovial fibroblasts were cultured in chondrocyte conditioned media which had been treated with anti-IL-6 antibody or anti-IgG antibody during immunoprecipitation. Depletion of IL-6 in the chondrocyte conditioned media ablated the ability of fibroblasts to secrete elevated IL-6 titres in response to chondrocyte conditioned media. Data are mean ± SEM. Statistical significance was determined by one-way ANOVA ***p < 0.001.

In order to determine if the cross-talk between chondrocytes and synovial fibroblasts is driven by IL-6 secreted from chondrocytes, we next depleted leptin-stimulated chondrocyte conditioned media of IL-6. This depletion of IL-6 from the chondrocyte conditioned media led to the ablation of cross-talk enhanced IL-6 secretion from synovial fibroblasts (p < 0.001) (Fig. 5). Importantly, there was no effect on the cross-talk enhanced IL-6 secretion from synovial fibroblasts when sham depletions were carried out using anti-IgG isotype control antibodies.

## Discussion

In this study we examined differences in pro-inflammatory cytokine signalling and adaptive immune cell populations in obese patients with hip OA, compared to normal-weight hip OA patients in order to better understand the impact of obesity on the pathology of OA. Our finding that both IL-6 and IL-8 were more abundant in the synovial fluid of obese hip OA patients, compared to normal-weight patients, supports the notion that increased adiposity is associated with a more inflammatory OA phenotype^2, 22, 23^ that may benefit from a different therapeutic approach.

In rheumatoid arthritis (RA) and in OA, cells of the adaptive immune system have been implicated in infiltrating the synovium, promoting the release of pro-inflammatory cytokines and maintaining a chronic inflammatory state within the joint^24, 25^. Therefore, we considered whether differences in either local or peripheral T lymphocyte subsets between obese and normal-weight patients could, in part, explain the differential synovial fluid cytokine profile observed between obese and normal-weight hip OA patients. However, our observation that there were no significant differences in T cell subsets in either the bone marrow or the peripheral blood between obese and normal-weight OA patients, suggested that these differential cytokine profiles between obese and normal-weight hip OA patients were unlikely to be mediated by differences in lymphocyte populations.

In addition to increased IL-6 we also found that the synovial fluid of obese patients exhibited, on average, greater levels of the soluble IL-6 receptor (sIL-6R). Importantly not all cell types, including synovial fibroblasts^26, 27^, express membrane bound IL-6R. Instead they are dependent on the soluble form of the receptor to bind to the IL-6 ligand and to form an active signalling complex with membrane bound gp130^27^. Therefore, the presence of greater amounts of sIL-6R together with IL-6 in obese hip OA synovial fluid is indicative of differential IL-6 signalling potential in this patient group.

In seeking to understand the cellular mechanism for these observations it was significant that primary synovial fibroblasts cultured from obese patients expressed significantly greater amounts of IL-6 mRNA, and secreted 2.5 fold more IL-6 than those cultured from normal-weight hip OA patients. Importantly, it is known that fibroblasts maintain their phenotype for up to 5 passages *in vitro* ^28^ and our findings here suggest that synovial fibroblasts from obese patients with hip OA are imprinted with a greater inflammatory phenotype. Unlike OA synovial fibroblasts, RA synovial fibroblasts are well characterised in this respect and are known to contribute to RA pathology through increased IL-6 expression under the drive of TNF-α^29–32^. Our data therefore lead us to pose the question of whether OA pathology in obese patients is much closer to that of RA due to the influence of excess adiposity and the adipokines secreted by this tissue.

Since the amount of IL-6 secreted from obese OA synovial fibroblasts was significantly lower than that found in the obese synovial fluid, we investigated whether stimulation of synovial fibroblasts with the adipokine leptin was required in order to better mimic the obese synovial fibroblast *in vivo* environment. Although leptin had no direct effect on inducing further IL-6 secretion from obese synovial fibroblasts, culturing synovial fibroblasts in conditioned media from leptin-stimulated chondrocytes resulted in a marked induction in the secretion of IL-6. Critically, this induction was significantly greater than that observed from leptin stimulation of chondrocytes, suggesting a synergistic activation of synovial fibroblasts via chondrocyte secreted IL-6. Such chondrocyte-fibroblast cross-talk has been suggested before, since it has previously been shown that TNFα release from chondrocytes induces catabolic gene expression from fibroblasts^33^.

**Figure 6:**
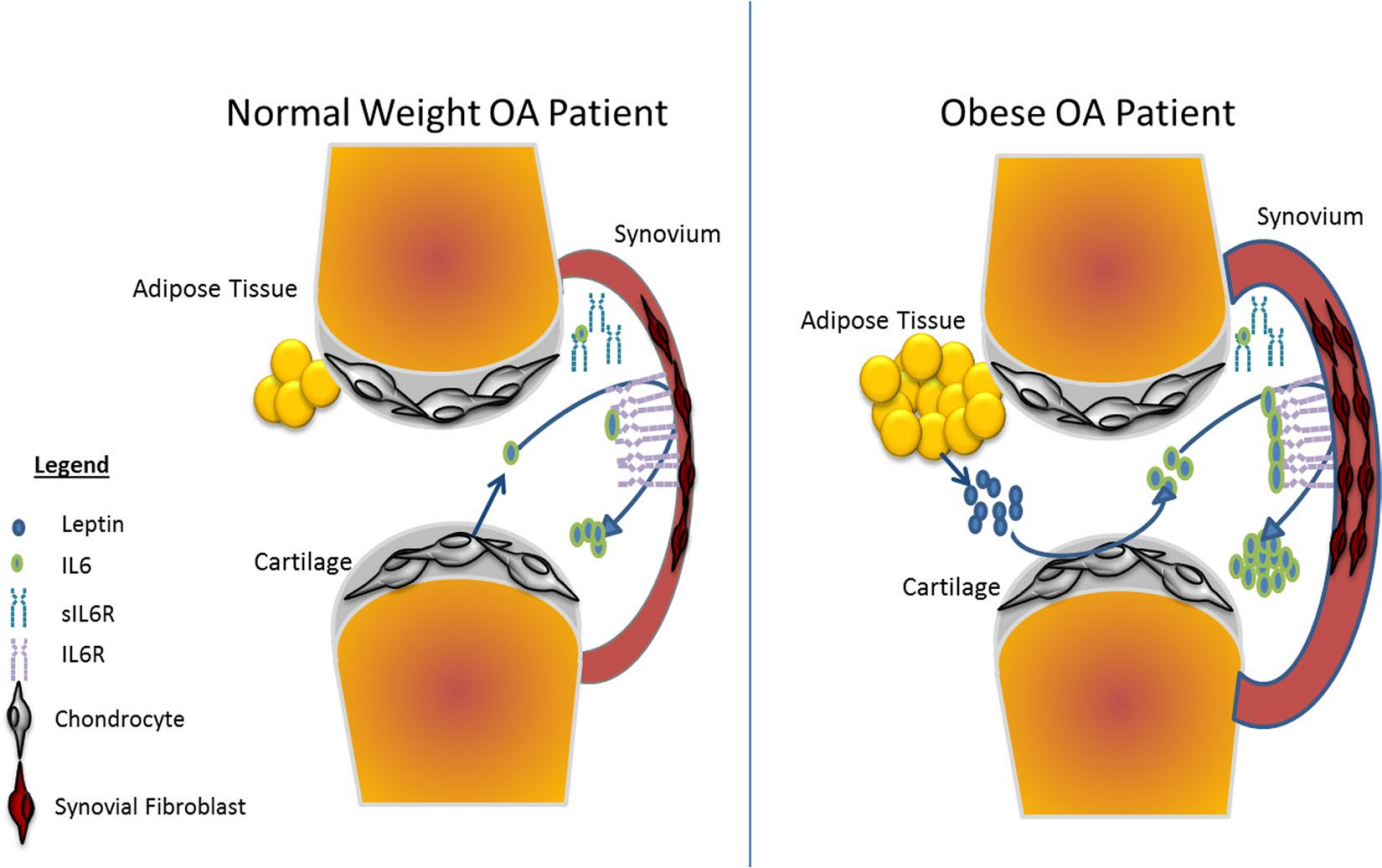
Predicted mechanism of IL-6 signalling via chondrocyte/synovial fibroblast cellular cross talk and its exacerbation in obese patients with OA. (**A**) In normal-weight OA patients, the chondrocytes in the articular cartilage secrete IL-6 into the synovial fluid. Chondrocyte secreted IL-6 binds to IL-6R on the surface of synovial fibroblasts and promotes IL-6 secretion from synovial fibroblasts. (**B**) In obese patients with OA, pathological levels of leptin stimulate greater secretion of IL-6 from the chondrocytes in the articular cartilage. This greater secretion of IL-6 promotes greater cellular cross-talk with synovial fibroblasts, leading to greater IL-6 secretion into the synovial fluid.

Since leptin induced no direct effect on IL-6 secretion from synovial fibroblasts, these data suggest that leptin is able to enhance cross-talk between synovial fibroblasts and chondrocytes, indirectly inducing secretion of greater IL-6 in synovial fibroblasts in an IL-6-dependent manner. This cross-talk mechanism may, therefore, account for the higher levels of IL-6 detected in the synovial fluids of obese patients with OA (Fig. 6).

Importantly, this is the first study to show that relatively modest IL-6 titres secreted from chondrocytes are able to drive vastly increased secretion of IL-6 from synovial fibroblasts. We hypothesise, therefore, that the tissues of the OA joint do not act independently in contributing to joint inflammation, but rather work in concert, with cellular cross-talk between cartilage and synovium driving an increase in IL-6 signalling. With this in mind, greater use of co-culture systems will be important in better modelling the cellular mechanisms of joint inflammation in human OA.

Furthermore, our data suggest IL-6 may play a significant role in the pathology of the OA joint in those patients who are obese. This may also have important implications for other joint pathologies such as ankylosing spondylitis^34^ and psoriatic arthritis^35^, where obesity is associated with increased risk and with poor clinical outcomes.

In addition, the leptin-enhanced synovial inflammation we report here provides further support for the metabolic (non-mechanical load) effect of obesity on joint disease, and could explain the association between joint disease and metabolic syndrome disorders such as hyperinsulinaemia and hypertension where elevated leptin levels are reported^36^. Finally, although our study did not explore the effect of leptin and obesity on osteoblast dysregulation in subchondral bone clearly future studies in this area are warranted, given the increasing evidence for the pathological role of adipokines in bone diseases^37^, and the evidence of subchondral bone changes in OA.

## Methods

### Patients

OA patients (n = 56) undergoing total hip replacement (THR) surgery at the Royal Orthopaedic Hospital, Birmingham, UK, and Russell’s Hall Hospital, Dudley, UK, were recruited to the study. Patients were divided based on BMI classification into either being of normal-weight (BMI 18.5–24.9, n = 19) or obese (BMI > 30, n = 27). The Kellgren Lawrence (K/L) system was used to classify the severity of OA severity. All patients had K/L grade 3 or 4, as determined by scoring of the patient’s x-ray radiographs by an orthopaedic surgeon prior to surgery. Diabetic patients and those who were taking anti-inflammatories within two weeks prior to arthroplasty surgery were excluded from the study. Informed consent was obtained from all patients prior to sample collection and ethical approval was granted by the Black Country REC (Ref. 10/1202/45). Sample collection, processing, storage and subsequent experimental procedures were carried out in compliance with Human Tissue Authority guidelines under the Human Tissue Act (2004). All patient identifiers were removed from the data and patient demographics are shown in Table 2.

**Table 2:** Patient Demographic Data.

	Normal Weight	Obese
	BMI 18–24.9 kg/m^2^	BMI < 30 kg/m^2^
Total	21	35
Age	66.9 ± 9.6	67.1 ± 8.7
Female	16 (50%)	16 (50%)
Male	5 (20.8%)	19 (79.16%)
BMI	22.98 ± 1.01	34.71 ± 3.84
Analgesic Use	47.60%	60%

### Isolation of primary cells and T lymphocytes

#### T cells

Peripheral blood was collected in heparin coated Vacutainers (BD). Peripheral blood mononuclear cells (PBMCs) containing T cell populations, were isolated by passing the blood through Ficoll-Paque (GE Healthcare) at 400 × *g* for 30 min. Following Buffy coat removal from the plasma-Ficoll interface, cells were washed once in phosphate buffered saline (PBS) before being frozen for use at a later time, or taken directly for use in flow cytometry analysis.

For the isolation of T cells from the bone marrow, bone chips from the arthritic hip were first incubated in 1 U/ml chromatographically purified collagenase CLSPA (Worthington Laboratories) for 1 h at 37 °C before being placed into a 1 ml pipette tip inside a 25 ml universal tube filled with culture media. Centrifugation (400 × *g* for 15 minutes) caused cellular material from within the bone to form a pellet in the bottom of the tube whilst keeping the bone collagenous material within the pipette tip. The pellet was resuspended in PBS and layered onto Ficoll-Paque as described above.

#### Primary OA Synovial Fibroblasts

Synovial membrane was excised and cut into small pieces of ~0.5 cm^2^. Five pieces of membrane were placed in a T25 culture flask (Corning) with complete fibroblast culture medium (RPMI 1640 supplemented with 10% FCS, 1% v/v non-essential amino acids, 1% v/v sodium pyruvate, 100 U/ml penicillin, 100 μg/ml streptomycin and 2 mM L-glutamine (Sigma Aldrich)). Tissue was cultured for 3–6 weeks until fibroblast outgrowth was observed, at which point tissue was removed. The phenotype of isolated synovial fibroblasts was first confirmed by positive CD55 expression by Western Blotting (Supplementary Fig. 1).

#### Primary OA Chondrocytes

Articular cartilage was removed from the bone using a scalpel and digested with collagenase 1 A (Sigma Aldrich) (1 mg/ml) for 6 h. Digested tissue was passed through a 70 µm cell strainer to remove debris and the flow-through was centrifuged at 2,000 × *g* for 5 min. The resulting cell pellet was resuspended in chondrocyte culture medium (DMEM supplemented with 10% FCS, 100 U/ml penicillin, 100 µg/ml streptomycin, 2 mM L-glutamine and 1% v/v non-essential amino acids (Sigma Aldrich)). The phenotype of the chondrocytes was confirmed by positive Type II collagen expression by Western Blotting (Supplementary Fig. 2).

### Cell stimulations and co-culture studies

Primary chondrocytes were stimulated with leptin at 20 ng/ml or 500 ng/ml in low-serum culture medium for 24 h in 6-well plates. The conditioned media was removed and transferred to wells containing primary synovial fibroblasts. The fibroblasts were cultured in the chondrocyte conditioned media for a further 24 h. The media was then removed and used in subsequent ELISA assays. Baseline non-stimulated conditioned media samples were also collected from both chondrocyte and fibroblast populations.

### IL-6 Depletion

IL-6 was depleted from the culture media using Dynabeads (ThermoFisher Scientific, Paisley, UK). Briefly, biotinylated anti-IL-6 or anti-IgG antibodies (both ThermoFisher Scientific) were incubated with conditioned cell culture supernatants overnight at 4 °C on a rotator. Dynabeads were incubated with antibody-treated supernatants at a concentration of 10 mg beads per 1 μg antibody for 30 minutes. Incubation with conditioned culture media from primary human chondrocytes for a further 30 minutes was followed by removal of the beads and captured IL-6 using a magnet. The IL-6 depleted conditioned media and control media (IgG) was collected and transferred immediately to primary human synovial fibroblasts for 24 hours.

### Determination of IL-6 secretion

Quantification of IL-6 production was determined using a commercially available ELISA (IL-6 DuoSet kit, R&D Systems) following the manufacturer’s instructions. Cell culture supernatants were diluted 1:200 with assay buffer before being read on a BioTek EL808 microtiter plate reader (BioTek, Swindon, UK).

### Determination of sIL-6R protein expression

Synovial fluid samples were collected, centrifuged at 3,000 × *g* to remove cell debris and frozen until required for Western blotting. Samples of equal protein load were then subjected to 12% SDS-PAGE, transferred to 0.2 µm PVDF membrane using a Trans-Blot Turbo (Bio-Rad, Hertfordshire, UK) and then immunoprobed with a sIL-6R antibody (1:1000 dilution; Abcam, Cambridge, UK), followed by a HRP linked anti-mouse secondary antibody (1:4000 dilution; GE Healthcare, Buckinghamshire, UK). Blots were then developed using Amersham ECL Prime (GE Healthcare, Buckinghamshire, UK), and imaged using a ChemiDoc MP System (Bio-Rad, Hertfordshire, UK).

### mRNA expression analysis

Total RNA was isolated from primary fibroblasts using TRIzol (ThermoFisher Scientific, Paisley, UK) according to the manufacturer’s instructions. Isolated RNA was resuspended in RNase-free water (GE Healthcare), quantified using a NanoDrop 2000 (Thermo Scientific) and qualitatively assessed using an Agilent Bioanalyzer (Agilent). RNAs with a RIN of >7 were used in quantitative real-time PCR.

Real-time PCR reactions were performed using QuantiFast SYBR RT-PCR reagents (Qiagen) and 5 μg total RNA. Primers for IL-6 (Forward: GCGCAGCTTTAAGGAGTTCCT; reverse: CCATGCTACATTTGCCGAAGA), IL-6R (Forward: TTCTTTATCAGGCTCTGAGTTCACA; reverse: GCGGCAACCAGTTTCCTTT) and gp130 (Forward: GGACTGACGGAACTTGGTGTCT; reverse: CACTTCGAGCACTGTCCAGTATTC) were designed using Primer Express 3 software (Applied Biosystems). All reactions were carried out using a LightCycler 480 II (Roche, Burgess Hill, UK).

### Bio-Plex Assays

Serum markers of inflammation were measured using an 8-plex Bio-Plex Assay (Bio-Rad, Hertfordshire, UK) comprising of IL-1β, IL-4, IL-6, IL-8, IL-10, IL-13, GM-CSF and TNF-α. The manufacturer’s instructions were followed throughout.

### *In situ* staining for β-galactosidase activity as a marker of cellular senescence

Primary chondrocytes, cultured in T25 culture flasks, were incubated with or without leptin (500 ng/ml) in low-culture media for 24 h, as previously described. As a positive control, a Human fibroblast cell line containing a 4-hydroxytamoxifen (4-OHT) sensitive inducible HRas oncogene was utilised, where senescence was induced by supplementation of the culture media with 333 nM of 4-OHT (Sigma Aldrich, UK).

Cellular senescence was determined using the Senescence Cells Histochemical Staining Kit (Sigma Aldrich, UK). In brief, cultured cells were washed twice in PBS (pH 7.4), fixed with 20% formaldehyde, 2% glutaraldehyde, 70.4 mM Na2HPO_4_, 14.7 mM KH2PO_4_, 1.37 M NaCl, and 26.8 mM KCl at room temperature for 7 min. Following PBS washes the cells were stained with 1 mg/ml of 1-X-gal (5-bromo-4-chloro-3-indolyl-bD-galactopyranoside), 5 mM potassium ferricyanide, 5 mM potassium ferrocyanide, and 2 mM MgCl_2_ in ddH_2_O. The plate was incubated at 37 °C for 3 hours and examined microscopically at ×10 magnification, and blue-stained cells were deemed to be senescent.

## Electronic supplementary material

Supplementary Information
