# Supplementary material for: IL-6 secretion in osteoarthritis patients is mediated by chondrocyte-synovial fibroblast cross-talk and is enhanced by obesity
Source: Sci Rep. 2017 Jun 14;7:3451. doi: 10.1038/s41598-017-03759-w (PMC5471184; doi:10.1038/s41598-017-03759-w)
Supplement: Supplementary file 1 — Supplementary Information [file 41598_2017_3759_MOESM1_ESM.pdf]

IL-6 secretion in osteoarthritis patients is mediated by chondrocyte-synovial fibroblast cross-talk and is enhanced by obesity

Mark J Pearson, Dietmar Herndler-Brandstetter, Mohammad A Tariq, Thomas A Nicholson, Ashleigh M Philp, Hannah L Smith, Edward T Davis, Simon W Jones and Janet M Lord

Supplementary Table S1: Cytokine expression in OA synovial fluid

**Cytokine**	**Abundance (pg/ml)**		***p* value**
	-leptin	***+leptin (500ng/ml)***	
IL4	0.41 ± 0.19	0.5 ± 0.25	0.7860
IL6	339.31 ± 6.18	691.19 ± 36.66	0.0007
IL8	566.03 ± 487.67	876.9 ± 755.5	0.7470
IL10	3.16 ± 0.49	3.08 ± 0.88	0.9407
IL17	21.01 ± 7.52	27.75 ± 13.53	0.6856
GM-CSF	55.53 ± 4.07	59.83 ± 3.68	0.4769
TNFα	15.26 ± 3.76	18.37 ± 5.69	0.6727

Statistical significance was determined by unpaired T test.

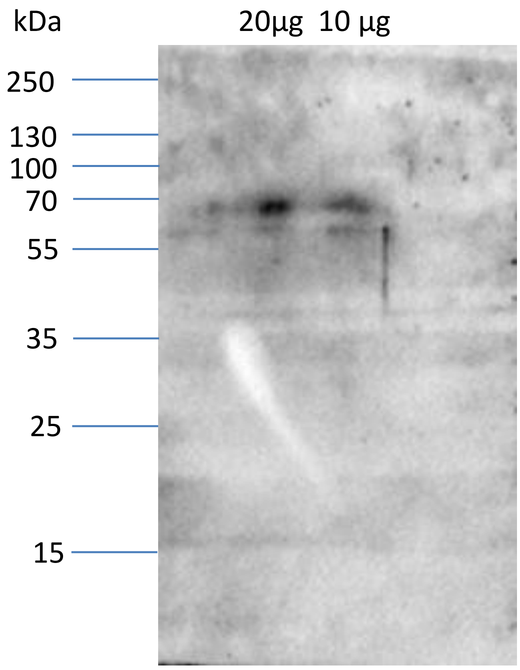

Supplementary Figure 1. Western blot demonstrating positive CD55 expression in primary OA synovial fibroblasts. Total protein RIPA lysates (20 and 10 µg) were subjected to 10% SDS-PAGE, and immunoprobed with a CD55 antibody (Abcam, ab133684). Blots were developed using ECL. Molecular weight marker used was PageRuler Plus (ThermoFisher).

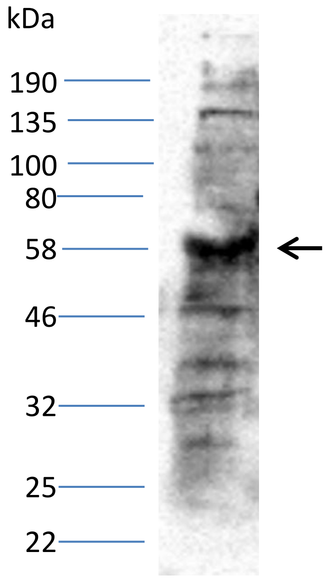

Supplementary Figure 2. Western blot demonstrating positive expression of Type II collagen in primary human OA chondrocytes isolated by collagenase digestion of articular cartilage. Total protein RIPA lysates (10 µg) were subjected to 10% SDS-PAGE, and immunoprobed with a Col2 antibody (Abcam, ab34712). Blots were developed using ECL. Molecular weight marker used was Colour Prestained Protein Standard, Broad-range (NEB).

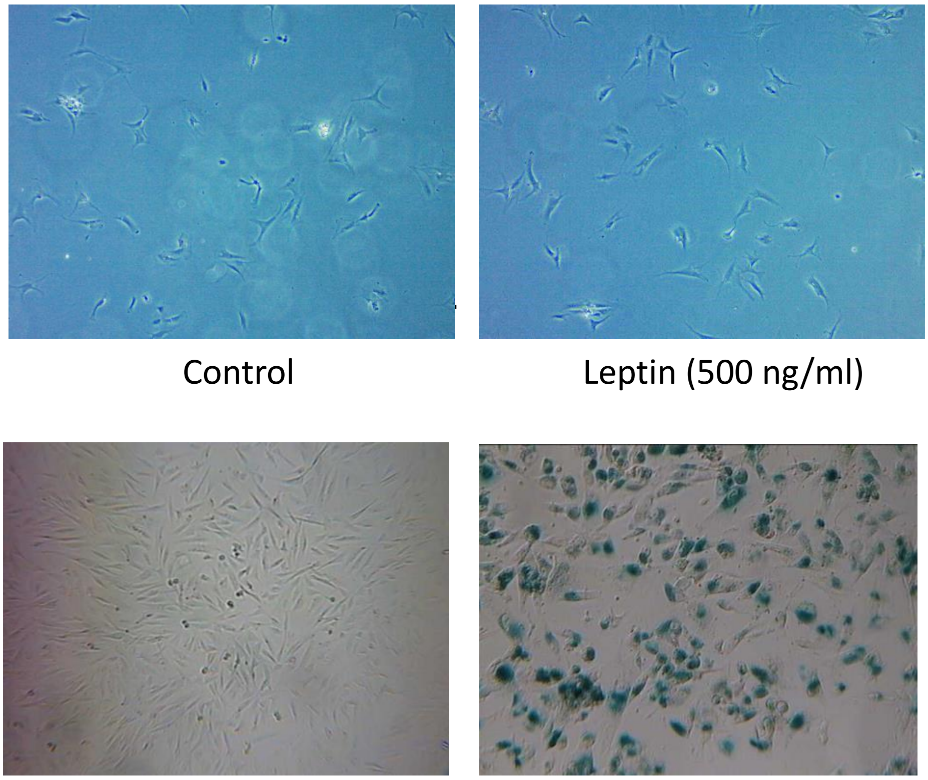

Supplementary Figure 3. ***In situ*** **staining for Beta-gal activity as a marker of cellular senescence.** Primary human OA chondrocytes cultured in T25 culture flasks were stimulated for 24h with leptin (500 ng/ml), or left unstimulated. Beta-gal staining was performed using a histochemical staining kit (Sigma Aldrich, UK). A HRas fibroblast cell line was used as a positive control, whereby senescence was induced by addition of 4-OHT. Images are at 4x magnification
